# A Pathogen’s Whisper: Reactivation of Quiescent Membranous Nephropathy by Disseminated Tuberculosis

**DOI:** 10.7759/cureus.85663

**Published:** 2025-06-09

**Authors:** Athiphat Banjongjit, Veerapat Wattanasatja, Suwasin Udomkarnjananun, Talerngsak Kanjanabuch

**Affiliations:** 1 Nephrology Unit, Department of Medicine, Vichaiyut Hospital, Bangkok, THA; 2 Department of Internal Medicine, Sunpasitthiprasong Hospital, Ubon Ratchathani, THA; 3 Center of Excellence Renal Immunology and Renal Transplantation, Faculty of Medicine, Chulalongkorn University, Bangkok, THA; 4 Center of Excellence in Kidney Metabolic Disorders, Faculty of Medicine, Chulalongkorn University, Bangkok, THA; 5 Division of Nephrology, Department of Medicine, Faculty of Medicine, Chulalongkorn University, Bangkok, THA; 6 Peritoneal Dialysis Excellence Center, King Chulalongkorn Memorial Hospital, Bangkok, THA

**Keywords:** autoimmune disease, glomerulonephritis, membranous nephropathy, nephrotic syndrome, pla2r, relapse, tuberculosis

## Abstract

Primary membranous nephropathy (MN) is an autoimmune glomerular disease commonly associated with anti-PLA2R antibodies, with relapses typically attributed to spontaneous immune reactivation. We report the first documented case of a relapse of primary, PLA2R-positive MN that was temporally and immunologically linked to disseminated tuberculosis (TB) infection. A 42-year-old man, previously in complete remission, developed severe nephrotic syndrome and acute kidney injury unresponsive to standard immunosuppressive regimens. Concomitant diagnosis of miliary TB was confirmed by culture and imaging. Remarkably, the MN relapse resolved completely with anti-TB therapy alone, without further immunosuppression, and remission has been sustained for over two years. This case highlights infection, specifically TB, as a modifiable and overlooked trigger of MN relapse, potentially via molecular mimicry or system immune activation. In TB-endemic regions, identifying infectious triggers early in relapsing MN may spare patients from unnecessary immunosuppression and facilitate long-term remission through targeted antimicrobial therapy.

## Introduction

Membranous nephropathy (MN) is the leading cause of non-diabetic nephrotic syndrome (NS) in adults, characterized histologically by subepithelial immune complex deposition and thickening of the glomerular basement membrane (GBM) [1-3]. It is primarily an autoimmune disease mediated by circulating autoantibodies targeting podocyte-expressed antigens such as phospholipase A2 receptor (PLA2R), thrombospondin type-1 domain-containing 7A (THSD7A), and neural epidermal growth factor-like 1 protein (NELL-1). These autoantibodies activate the complement system, leading to podocyte injury via the membrane attack complex (MAC), and resulting in proteinuria [4,5].

Approximately 75-80% of MN cases are classified as primary, while secondary MN arises in association with systemic lupus erythematosus, malignancies, or chronic infections including hepatitis B and C, malaria, syphilis, and leprosy [1]. Although *Mycobacterium tuberculosis* (TB) has been recognized as a rare infectious cause of secondary MN, its role as a potential trigger for the relapse of primary MN remains poorly understood and rarely reported [6,7].

Relapses of primary MN are not uncommon and are often attributed to spontaneous reactivation of immunologic processes [8]. However, infections have increasingly been identified as potential external triggers of disease flares, even in previously stable cases. In particular, systemic infections may provoke autoimmune responses through mechanisms such as molecular mimicry and systemic immune stimulation, including patients with anti-PLA2R positivity [9-11].

Here, we report what appears to be the first documented case of a relapse of primary, PLA2R-positive MN temporally and immunologically linked to disseminated TB infection. The patient’s NS was refractory to standard immunosuppressive therapy but resolved completely following anti-tuberculosis treatment alone. This case not only underscores the critical importance of evaluating for infectious triggers in MN relapse, particularly in TB-endemic regions, but also highlights the complex interplay between infection and autoimmunity in glomerular diseases.

## Case presentation

A 42-year-old male healthcare worker with no prior comorbidities was incidentally diagnosed with hypertension and proteinuria during a routine medical screening. Initial investigations revealed 3+ proteinuria with 24-hour urinary protein excretion of 2.5 g/day. Kidney function was preserved (serum creatinine 0.9 mg/dL, estimated glomerular filtration rate (eGFR) 133 mL/min/1.73 m², calculated using the Thai eGFR equation as recommended for the local population [12]), and serum albumin was mildly reduced at 3.3 g/dL. The patient was started on enalapril 40 mg/day, but proteinuria persisted over a two-year period, fluctuating between 0.7 and 1.3 g/day despite conservative therapy.

Approximately six years before the diagnosis of TB, he was hospitalized for progressive generalized edema, foamy urine, and uncontrolled blood pressure. Laboratory evaluation showed 24-hour urinary protein of 3.7 g/day and serum albumin of 3.3 g/dL. Viral serologies (hepatitis B virus (HBV), hepatitis C virus (HCV), and HIV), antinuclear antibody (ANA), and serum protein electrophoresis were negative. There was no evidence of light chain restriction. Serum complement levels were within normal range (C3 97.5 mg/dL, C4 20.9 mg/dL, CH50 28.1 U/mL). Kidney ultrasound showed normal-sized kidneys with preserved cortical echotexture (right kidney: 11.7 × 4.0 cm; left kidney: 11.6 × 5.0 cm), and no evidence of structural abnormalities.

Kidney biopsy revealed classical histopathologic features of MN, including diffuse capillary wall thickening, spike formation on silver staining, and numerous subepithelial electron-dense deposits. Immunofluorescence microscopy showed strong fine granular IgG and C3 deposition along the outer aspect of the GBM without C1q or mesangial deposits, consistent with primary MN. Electron microscopy further demonstrated absence of subendothelial and mesangial immune deposits and no evidence of tubuloreticular structures (TRS), reinforcing the diagnosis of primary MN. Anti-PLA2R staining was not performed due to a limited kidney biopsy specimen. However, the diagnosis of primary MN was supported by a positive anti-PLA2R antibody assay. The patient received steroid, mycophenolate mofetil, and rituximab, achieving complete remission.

Three years prior to TB diagnosis, the patient relapsed with severe NS (proteinuria 14 g/day, serum albumin 2.1 g/dL) and acute kidney injury (serum creatinine 2.7 mg/dL). Repeat testing confirmed re-emergence of anti-PLA2R antibodies at a titer of 144 IU/mL. Immunosuppressive therapy was reinitiated with the Ponticelli regimen over six months, followed by a calcineurin inhibitor. However, nephrotic-range proteinuria persisted.

**Figure 1:**
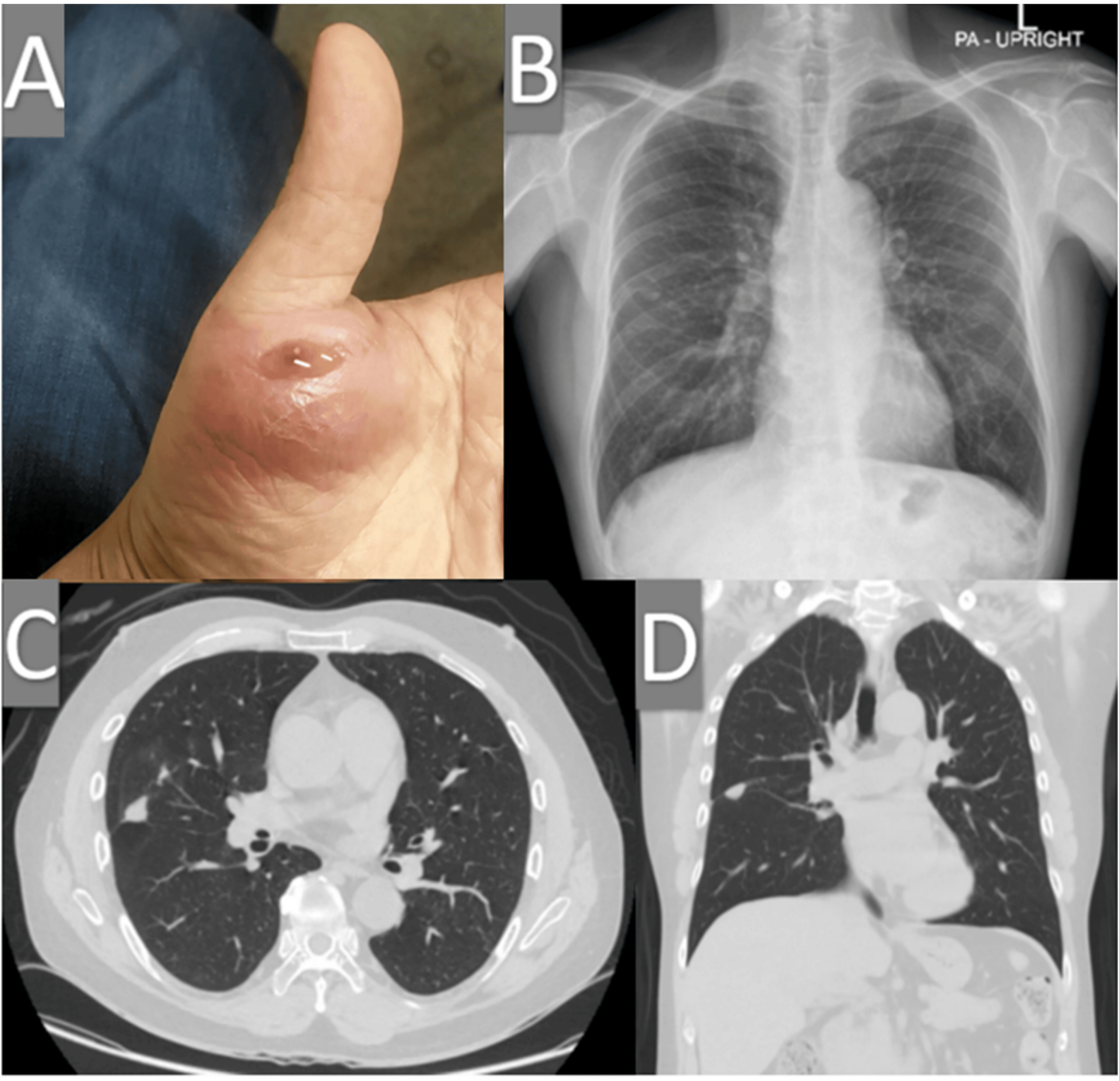
(A) Cutaneous abscess on the right thenar area with yellow pus accumulation. (B) Chest radiograph showing diffuse reticulonodular opacities with mass-like patchy infiltration in the right middle lung. (C, D) Chest CT images reveal a mass-like consolidation with surrounding centrilobular nodules and bronchiectasis in the lateral segment of the right middle lung.

At approximately Month 2 from TB diagnosis, the patient developed localized pain and swelling at the right metacarpophalangeal (MP) joint. Joint aspiration was negative for crystals and aerobic culture. The calcineurin inhibitor was tapered during this period due to suspected occult infection. Shortly thereafter, a fluctuant swelling developed over the right thenar eminence. Spontaneous drainage of purulent material at Month 0 tested positive for acid-fast bacilli on Ziehl-Neelsen staining, and *M. tuberculosis* was subsequently confirmed by culture (Figure 1A). Chest imaging revealed bilateral miliary nodular opacities and consolidations, consistent with disseminated TB (Figure 1B-1D). Annual chest imaging is routinely performed as part of our GN follow-up protocol, and all prior imaging in this patient was negative.

**Figure 2:**
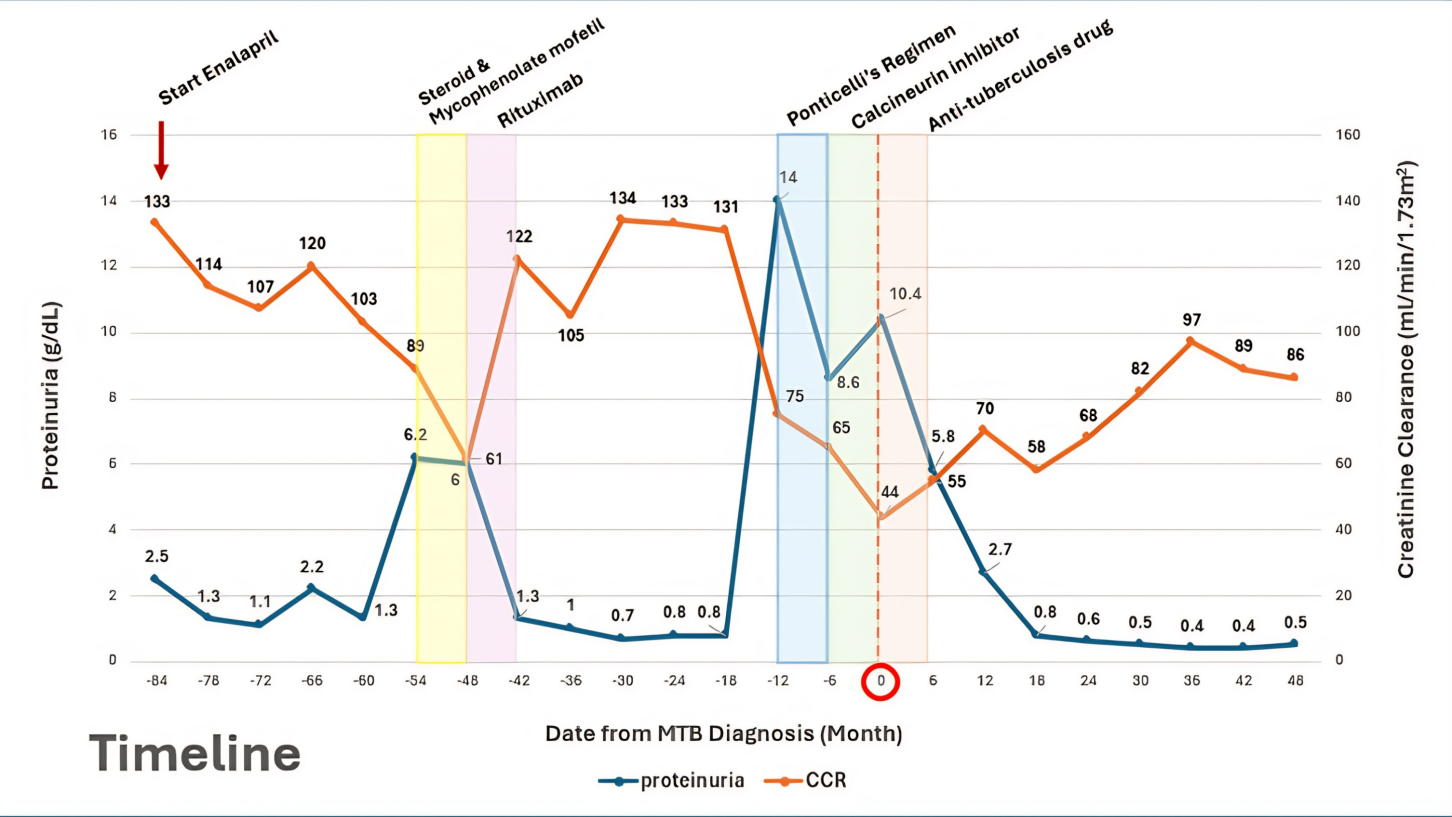
Timeline depicting the relapse of membranous nephropathy following Mycobacterium tuberculosis infection. CCR: creatinine clearance rate, MTB: Mycobacterium tuberculosis

Anti-tuberculosis therapy with a standard six-month regimen (2HRZE/4HR) was initiated immediately, and the calcineurin inhibitor was discontinued. Anti-PLA2R antibody titer at this time was 145.9 RU/mL. Over the following months, the patient exhibited marked clinical improvement. Proteinuria declined progressively from 10.4 g/day to 0.5 g/day by Month 24 and remained in sustained remission through Month 48. Kidney function improved in parallel. Anti-PLA2R titers also declined steadily: 76.7 RU/mL at Month 6, 13.7 at Month 11, 16.2 at Month 12, and <2 RU/mL from Month 24 onward. While a delayed response to the Ponticelli regimen cannot be fully excluded, the absence of clinical or serologic improvement during active immunosuppression and the sustained remission following anti-TB therapy suggest a contributory role of disseminated TB in this relapse. The patient has remained in complete remission for over two years without further immunosuppressive therapy. Figure 2 illustrates the clinical timeline.

## Discussion

This case illustrates how disseminated TB can trigger a relapse of previously quiescent primary MN. Our patient, who had achieved complete remission of PLA2R-positive MN with immunosuppressive therapy, presented with a relapse temporally associated with the onset of miliary TB. The relapse was marked by severe nephrotic syndrome, hypoalbuminemia, and acute kidney injury, and proved resistant to standard immunosuppressive regimens. Notably, there was no clinical or serological improvement during active immunosuppression, and full remission was achieved only after initiating anti-TB treatment, without further immunosuppressive therapy, supporting a strong temporal and mechanistic association between TB and MN relapse.

While relapses of primary MN are often attributed to spontaneous immune reactivation [1], infections have increasingly been recognized as potential external triggers [13,14]. Tuberculosis, although a known cause of secondary MN, is rarely implicated in reactivation of PLA2R-positive disease [15]. This case appears to be one of the first to document disseminated TB precipitating a relapse of primary MN with high anti-PLA2R titers that was refractory to standard immunosuppression but reversed with antimicrobial therapy alone.

**Figure 3:**
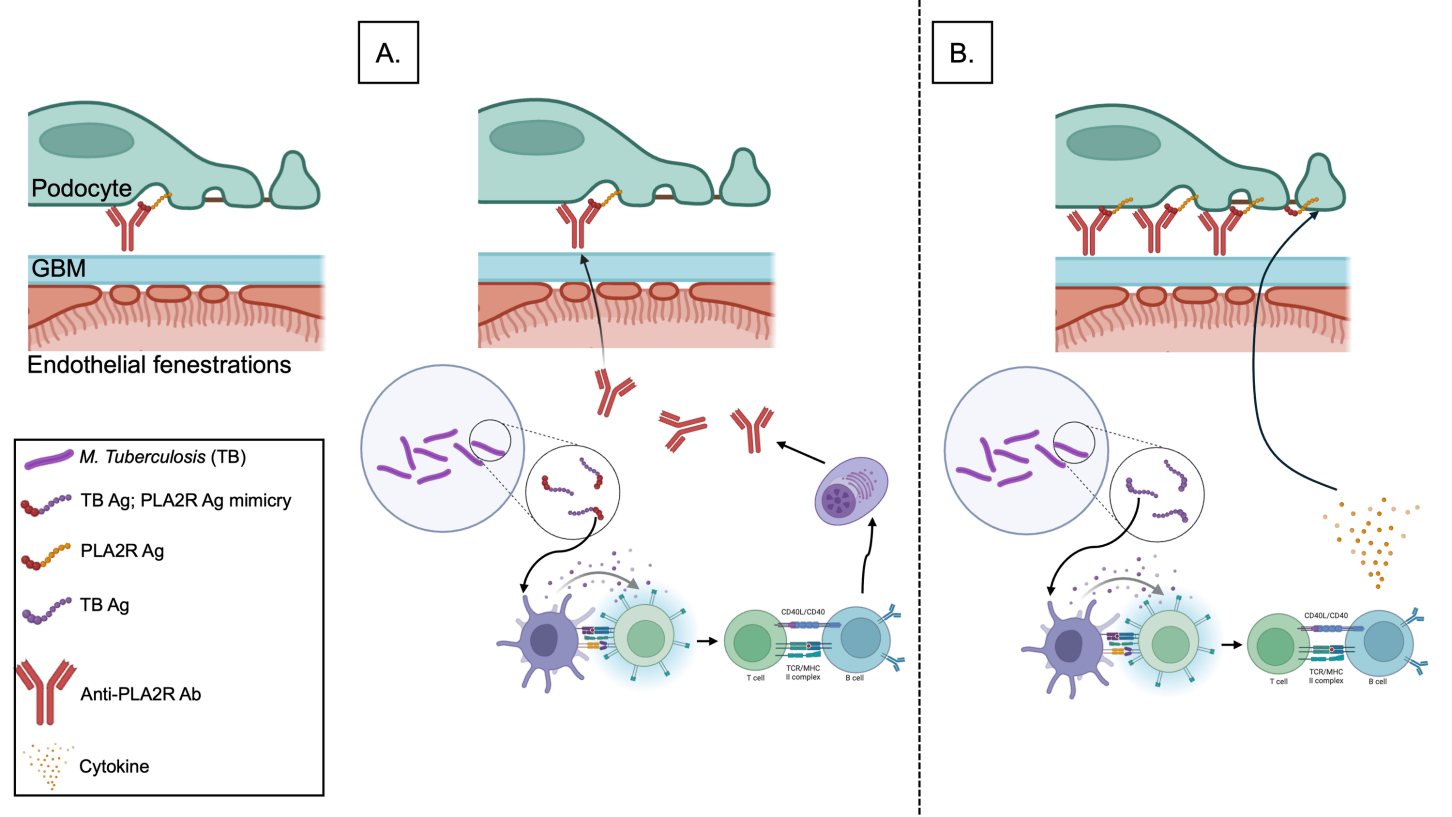
Proposed Mechanisms of Mycobacterium tuberculosis (TB)–Associated Membranous Nephropathy. (A) Molecular mimicry: TB surface antigens may share conformational epitopes with PLA2R, leading to a breakdown in immune tolerance and the production of cross-reactive anti-PLA2R autoantibodies. (B) Systemic immune activation: Disseminated TB infection provokes a cytokine surge and enhances antigen presentation, potentially reactivating latent autoimmune pathways and triggering relapse in PLA2R-associated membranous nephropathy Figure created with BioRender.com. GBM: glomerular basement membrane, PLA2R: phospholipase A2 receptor, Ab: antibody, Ag: antigen

The underlying mechanisms may include molecular mimicry, where mycobacterial antigens resemble PLA2R epitopes and provoke a cross-reactive autoimmune response [16]. Alternatively, systemic immune activation through cytokine release and enhanced antigen presentation in the setting of disseminated TB may rekindle a previously dormant autoimmune process (Figure 3) [17]. This is supported by the observed anti-PLA2R antibody titers trend: 145.9 RU/mL at TB diagnosis, with a progressive decline to <2 RU/mL over 24 months following anti-TB treatment, mirroring clinical remission. Similar patterns have been reported in infection-associated MN relapses following COVID-19 [13].

TB-associated MN remains exceptionally rare, with most reported cases describing secondary forms of MN that respond to TB treatment [18]. To our knowledge, PLA2R-positive MN relapsing in the context of active TB - with no response to sequential immunosuppressive regimens - has not been previously described. The failure to respond to immunosuppressive therapy further underscores the importance of identifying and treating underlying infections in relapsing glomerular diseases [19].

Kidney imaging in this case was unremarkable, and chest imaging had been routinely performed prior to the relapse, with no prior abnormalities - highlighting the abrupt onset and disseminated nature of TB at relapse. This reinforces the clinical value of infection surveillance, even in the absence of prior imaging findings. In TB-endemic regions, clinicians should maintain a high index of suspicion for occult infections in patients presenting with unexpected or refractory MN relapses. Early recognition is essential to avoid inappropriate escalation of immunosuppression, which may otherwise worsen both kidney and infectious outcomes [14].

## Conclusions

This case highlights disseminated TB as a potential immunologic trigger for relapse of PLA2R-positive MN. The patient’s complete and sustained remission following anti-TB therapy alone, despite prior immunosuppressive failure, highlights the need for infection surveillance in glomerular disease management. Recognizing infection as a modifiable trigger of autoimmune activation may enhance treatment precision, reduce immunosuppressive exposure, and improve long-term outcomes.
